# Multimodal Medical Image Fusion by Adaptive Manifold Filter

**DOI:** 10.1155/2015/564748

**Published:** 2015-11-18

**Authors:** Peng Geng, Shuaiqi Liu, Shanna Zhuang

**Affiliations:** ^1^School of Information Science and Technology, Shijiazhuang Tiedao University, Shijiazhuang 050043, China; ^2^College of Electronic and Information Engineering, Hebei University, Baoding 071000, China

## Abstract

Medical image fusion plays an important role in diagnosis and treatment of diseases such as image-guided radiotherapy and surgery. The modified local contrast information is proposed to fuse multimodal medical images. Firstly, the adaptive manifold filter is introduced into filtering source images as the low-frequency part in the modified local contrast. Secondly, the modified spatial frequency of the source images is adopted as the high-frequency part in the modified local contrast. Finally, the pixel with larger modified local contrast is selected into the fused image. The presented scheme outperforms the guided filter method in spatial domain, the dual-tree complex wavelet transform-based method, nonsubsampled contourlet transform-based method, and four classic fusion methods in terms of visual quality. Furthermore, the mutual information values by the presented method are averagely 55%, 41%, and 62% higher than the three methods and those values of edge based similarity measure by the presented method are averagely 13%, 33%, and 14% higher than the three methods for the six pairs of source images.

## 1. Introduction

With the development of medical technology, computer science, and biomedical engineering technology, the medical image technology can provide the clinical diagnosis with a variety of multimodal medical images such as the computed tomography (CT), the magnetic resonance imaging (MRI), the single photon emission computed tomography (SPECT), the positron emission tomography (PET), and ultrasonic images [1]. Different medical image can display different information of the same viscera in the body. For example, the MRI is good at expressing the soft tissue information compared to the CT. However, the CT image can provide better information of tissue calcification and bone segment than the MRI can. In the clinic application, a single modal medical image often cannot provide doctors with enough information to make the correct diagnosis [2, 3]. It is necessary to combine different modal images into one image with enough information of source images. The fused medical images can contain the vital information from the several modal images to demonstrate the comprehensive information of diseased tissue or organs. At the same time, the redundant information in the source images is abrogated. Hence, the doctor can easily make an accurate diagnosis or determine the accurate therapeutic scheme.

Generally, medical image fusion algorithms are divided into two categories: spatial domain methods and multiscale decomposition domain methods [4]. The spatial domain methods combine pixels or regions from source images into fused images in the spatial domain [5]. The other methods adopt the sparse transforms such as traditional wavelets pyramid, contourlet [6], and nonsubsampled contourlet transform [6]. Compared with the spatial domain methods, multiscale decomposition domain methods are of more time complexity because of their redundancy decomposition, especially for nonsubsampled contourlet transform-based fusion approaches. On the other hand, the spatial domain methods can be introduced into the clinical application and surgery procedure because of the low complexity. Generally speaking, fusion methods based on spatial domain can be performed in real time to provide clinic doctor with real-time diagnosis in the surgery. Therefore, this paper focuses on the multimodal medical image fusion method in the spatial domain.

In the latest years, many edge-preserving are active research topic in image processing such as the bilateral filter, weighted least squares [7], guided filter [8], domain transform filter [9], and cost-volume filter [10]. Due to the fact that edge-preserving filters can avoid ringing artifacts and preserve well the edge structure information, these edge-preserving filters have already been widely used in image matching, image dehazing, image denoising, and image classification [11]. The guided filter assumes that the filtered output is a linear transformation of the guidance image. Owing to guided filter based on a local linear model, Kang [11] introduced firstly the guided filter into image fusion area in spatial domain. The domain transform filter preserves the geodesic distance between points on the curve, adaptively warping the input signal so that 1D edge-preserving filtering can be efficiently performed in linear time. The recursive filter used in the domain transform filter makes itself not effective to deal with the complex edge structure with a large amount of discontinuity area. The cost-volume filter is a discrete optical flow approach which handles both fine (small-scale) motion structure and large displacements. The cost-volume leads to generic and fast framework that is widely applicable to computer vision problems. The adaptive manifold filter [12], which has the advantages of better global diffusion and edge-preserving ability, is a real-time high dimension filter on the basis of iterative filter. Moreover, adaptive manifold filter can produce high-quality results and require less memory. In this paper, the adaptive manifold filter is firstly introduced into the images fusion area, especially the multimodal medical image fusion.

## 2. Methods

### 2.1. Adaptive Manifold Filter

The adaptive manifold filter is the first high-dimensional filter for performing high-dimensional filtering of images and videos in real time [13]. The adaptive manifold filter is quite flexible and capable of producing responses that approximate to either standard Gaussian filters or non-local-means filters. The process of the adaptive manifold filter can mainly be divided into three parts: the projection part, the blurring part, and the gathering part.

Let *S* ⊂ *R* ^*d*_*S*_^ → *R* ⊂ *R* ^*d*_*R*_^ be a signal associating each point from its *d*_*S*_-dimensional spatial domain *S* to a value in its *d*_*R*_-dimensional range *R*. With regard to gray image, *d*_*S*_ and *d*_*R*_ are equal to 2 and 1, respectively [14].

Then, the number of manifolds *K* is independent of the filter dimensionality and can be generated by the following function:

(1) $$\begin{array}{c} K=2+\max\left(\;2,\left[\;H_{S}L_{R}\;\right]\;\right), \end{array}$$

where *L*_*R*_ is defined as a linear correction calculated from the range standard deviation and *H*_*S*_ defines the height calculated from the spatial standard deviation. Let {*P*_1_,…, *P*_*N*_} be the set of *N* samples obtained by sampling *S* using a regular grid. We refer to each *p*_*i*_ as a pixel. *k*th *d*_*S*_-dimensional adaptive manifold can be described by a graph (*p*_*i*_, *η*_*ki*_), and the manifold value *η*_*ki*_ ∈ *R* associated with pixel *p*_*i*_ ∈ *S* is defined by the evaluation of a function *η*_*ki*_ : *S* → *R* at *p*_*i*_ : *η*_*ki*_ = *η*_*ki*_(*p*_*i*_) [15]. When the low-pass filtering is performed over the input signal *f*, the first manifold *η*_1_ can be generated:

(2) $$\begin{array}{c} \eta_{1}\left(\;p_{i}\;\right)=\left(\;h_{\sum S}*f\;\right)\left(\;p_{i}\;\right), \end{array}$$

where *∗* is convolution operation and *h*_∑*S*_ is a low-pass filter with covariance matrix ∑*S*. Based on the first manifold *η*_1_, Gaussian distance-weighted projection of the pixel values of the image is performed on the manifold. The projection process can be represented as

(3) $$\begin{array}{c} \Psi_{1}\left(\;{\hat{\eta }}_{ki}\;\right)=\phi_{\sum R/2}\left(\;\eta_{ki}-f_{i}\;\right)f_{i}, \end{array}$$

where ∑*R*/2 is diagonal covariance matrix with size of *d*_*R*_ × *d*_*R*_ which controls the decay of the Gaussian kernel *ϕ*. Gaussian filtering is performed over each manifold mixing the values Ψ_1_ from all sampling points ${\hat{\eta }}_{ki}$. Mathematically, the blurred values $\Psi_{2}\left({\hat{\eta }}_{ki}\right)$ can be expressed as

(4) Ψ2η^ki=∑pi∈Sϕ∑ηη^ki−p^ifi,∑η=∑S00∑R2,

where ${\hat{p}}_{j}=\left(p_{j},f_{j}\right)$ and Ψ_2_ is the Gaussian filtering on *d*-dimensional space. The final filter response *g*_*i*_ for each pixel is generated by interpolating blurred values Ψ_2_ gathered from all adaptive manifolds:

(5) $$\begin{array}{c} g_{i}=\frac{\sum_{k=1}^{K}\omega_{ki}\phi_{blur}\left({\hat{\eta }}_{ki}\right)}{\sum_{k=1}^{K}\omega_{ki}\phi_{blur}^{0}\left({\hat{\eta }}_{ki}\right)},\;\omega_{ki}=\phi_{\sum R/2}\left(\eta_{ki}-f_{i}\right), \end{array}$$

where *K* is the total number of adaptive manifolds that will be used to filter a signal *f* and *ω*_*ki*_ is the weight corresponding to *K*.

### 2.2. Modified Local Contrast

The contrast feature of image can evaluate the difference of the intensity value at some pixels around the neighbor pixels. The human visual system is highly sensitive to the intensity contrast rather than the intensity value itself. In general, the same intensity value looks like a different intensity value depending on intensity values of neighboring pixels. According to [16], local luminance contrast can be defined as follows:

(6) $$\begin{array}{c} C=\frac{L-L_{B}}{L_{B}}=\frac{L_{H}}{L_{B}}, \end{array}$$

where *L* is the local brightness of image and *L*_*B*_ is the brightness of the local background. In general, *L*_*B*_ is regarded as local low-frequency information of an image and *L*_*H*_ is treated as local high-frequency information of an image. Hence, a proper way to select high-frequency and low-frequency information is necessary to ensure better information interpretation. The modified spatial frequency (MSF) [17] is calculated according to the row frequency, column frequency, and diagonal frequency of the image. The larger modified spatial frequency leads to the salient features such as edges, lines, and region boundaries. Hence, the modified spatial frequency of an image can be used as the high-frequency information of the image. On the other side, the filtered result of an image by adaptive manifold filter can be used as the low-frequency information of the image. Mathematically, the modified local contrast MLC(*i*, *j*) in spatial domain is given by

(7) $$\begin{array}{c} MLC\left(\;i,j\;\right)=\left\{\begin{array}{cc} \frac{MSF\left(i,j\right)}{AMF\left(i,j\right)}, & \text{if }AMF\left(i,j\right)\neq 0,\\ MSF\left(i,j\right), & \text{if }AMF\left(i,j\right)=0, \end{array}\right. \end{array}$$

where MSF(*i*, *j*) is the modified spatial frequency of image *I* at *i* row and *j* column. On the other hand, AMF(*i*, *j*) is the filtered result of image *I*(*i*, *j*) by adaptive manifold filter. The modified spatial frequency is capable of capturing the fine details presented in the image because of incorporating the diagonal frequency, the row frequency, and column frequency. The modified spatial frequency can be calculated as

(8) $$\begin{array}{c} MSF\left(\;i,j\;\right)=\sqrt{{SF}^{2}\left(i,j\right)+{DF}^{2}\left(i,j\right)}, \end{array}$$

where the spatial frequency SF(*i*, *j*) can be calculated as follows [18, 19]:

(9) SFi,j=1MN∑i=1M ∑j=2NIi,j−Ii,j−12+Ii,j−Ii−1,j2,

where *M* and *N* denote the number of row and column of image *I*(*i*, *j*), respectively. The diagonal frequency DF(*i*, *j*) can be expressed as

(10) $$\begin{array}{c} DF\left(\;i,j\;\right)=\sqrt{\frac{1}{MN}\overset{M}{\underset{i=1}{\sum }}\;\overset{N}{\underset{j=1}{\sum }}{\left(I\left(i,j\right)-I\left(i-1,j-1\right)\right)}^{2}+{\left(I\left(i-1,j\right)-I\left(i,j-1\right)\right)}^{2}}. \end{array}$$

### 2.3. Summary of Fusion Method

**Figure 1:**
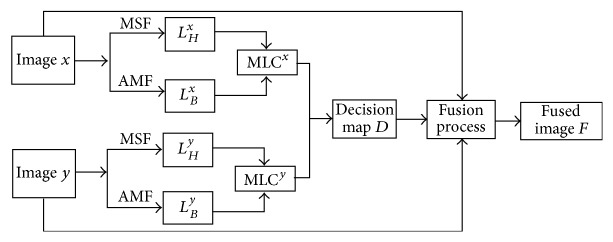
Schematic diagram of proposed fusion algorithm.

Figure 1 demonstrates the schematic diagram of proposed fusion algorithm. The steps of the proposed fusion approach in this paper can be briefly summarized as the following five steps:

 (1) The source medical images *x* and *y* are registered, respectively.
 (2) The source medical images *x* and *y* are filtered by the adaptive manifold filter to obtain *L*_*B*_^*x*^(*i*, *j*) and *L*_*B*_^*y*^(*i*, *j*) as the low-frequency part of modified local contrast information:
  (11) LBxi,j=AMFIxi,j,LByi,j=AMFIyi,j.
 (3) The modified spatial frequency of source medical image is adopted as the high-frequency information of modified local contrast information according to (7). The modified local contrast of source images *x* and *y* can, respectively, be defined as MLC^*x*^(*i*, *j*) and MLC^*y*^(*i*, *j*) which are expressed as
  (12) MLCxi,jLHxLBx=MSFxi,jAMFxi,j,MLCyi,jLHyLBy=MSFyi,jAMFyi,j,
 where *L*_*H*_^*x*^ and *L*_*H*_^*y*^ are equal to MSF^*x*^(*i*, *j*) and MSF^*y*^(*i*, *j*) and represent the high-frequency information of modified local contrast information, respectively.
 (4) The decision map *D*(*i*, *j*) can be expressed as follows to fuse the source multimodal medical images:
  (13) $$\begin{array}{c} D\left(\;i,j\;\right)=\left\{\begin{array}{cc} 1, & \text{if }{MLC}^{x}\left(i,j\right)\geq {MLC}^{y}\left(i,j\right),\\ 0, & \text{other.} \end{array}\right. \end{array}$$
 (5) Finally, the fused medical image *F*(*i*, *j*) can be merged by the decision map:
  (14) $$\begin{array}{c} F\left(\;i,j\;\right)=I^{x}\left(\;i,j\;\right)*D\left(\;i,j\;\right)+I^{y}\left(\;i,j\;\right)*\bar{D\left(i,j\right)}. \end{array}$$

## 3. Results

### 3.1. Experimental Setup

**Figure 2:**
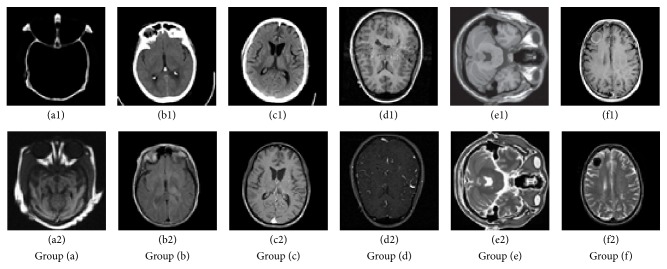
Several kinds of multimodal medical images.

To evaluate the performance of the proposed fusion method, experiments have been performed on six pairs of images shown in Figure 2, respectively. These images are characterized in four different categories: (1) CT and MRI, (2) T1-weighted MRI (T1-MRI) and T2-weighted MRI (T2-MRI), (3) MRI and magnetic resonance angiography (MRA) images, and (4) Gadolinium-Diethylenetriamine Pentaacetic Acid MRI (GD-MRI) and T1-weighted MRI. Groups (a) and (b) in Figure 2 are CT images and MRI whereas groups (e) and (f) in Figure 2 are T1-MRI and T2-MRI, respectively. Group (c) in Figure 2 is the T1-MRI and GD-MRI images, respectively. Group (d) in Figure 2 is the T1-MRI and MRA, respectively. The corresponding pixels of two input images have been perfectly matched. All images have the same size of 256 × 256 pixel, with 256-level gray scale. On the one hand, the proposed method is compared with some classic image fusion methods such as principal components analysis (PCA), Laplacian pyramid, Gradient pyramid, and shift invariant discrete wavelet transform (SIDWT) which are compared in many works [4, 20]. On the other hand, the performance of the proposed method is compared with the modified spatial frequency of NSCT coefficients motivated PCNN method proposed by Sudeb [17] and the dual-tree complex wavelet transform method combined with the nonsubsampled direction filter bank (NSDFB) by Liu [21]. In Sudeb's scheme based on NSCT, the pyramid filter and the direction filter are set to “pyrexc” and “vk,” respectively. The decomposition levels of NSCT are set to [1, 2, 4] in accord with [17]. The three levels of dual-tree complex wavelet transform are adopted to decompose the NSDFB coefficients in Liu's method. The direction filter is set to “cd.” Furthermore, the guided filter method in spatial domain proposed by Kang [11] is compared with the proposed method because the proposed fusion method is part of the spatial-based domain fusion method. In Kang's method, the source images are decomposed into a base layer and a detail layer by average filtering. The guided filtering-based weighted average technique is adopted to make full use of spatial consistency for fusion of the base and detail layers. The parameters used in [11] are directly adopted in this comparison. The filter spatial standard deviation and filter range standard deviation is set to 14 and 0.10 in the adaptive manifold filter, separately.

### 3.2. Evaluation Metrics

#### 3.2.1. Mutual Information

Mutual information (MI), proposed by Piella [22], can demonstrate how much information the fused image conveys about the reference image. The MI is defined as MI = MI_*xF*_ + MI_*yF*_, where MI_*tF*_ can be calculated by

(15) $$\begin{array}{c} MI\left(\;t:F\;\right)=\overset{L}{\underset{u=1}{\sum }}\;\overset{L}{\underset{v=1}{\sum }}h_{t,F}\left(\;u,v\;\right)\log_{2}\frac{h_{t,F}\left(u,v\right)}{h_{t}\left(u\right)h_{F}\left(v\right)}, \end{array}$$

where *t* and *F* denote the source image (*x* or *y*) and fused image, respectively. *h*_*t*,*F*_ is the joint gray level histogram of *t* and *F*, *h*_*t*_ and *h*_*F*_ are the normalized gray level histograms of *t* and *F*, and *L* is the number of bins. Hence, the larger MI value indicates that the fused image acquires more information from image *x* and image *y*.

#### 3.2.2. Edge Based Similarity Measure

The edge based similarity measure *Q* ^*AB*/*F*^ [23] gives the similarity between the edges transferred in the fusion process. Mathematically, *Q* ^*AB*/*F*^ is defined as

(16) $$\begin{array}{c} Q^{AB/F}=\frac{\sum_{i=1}^{M}\;\sum_{j=1}^{N}\left[Q_{i,j}^{xF}\omega_{i,j}+Q_{i,j}^{yF}\omega_{i,j}\right]}{\sum_{i=1}^{M}\;\sum_{j=1}^{N}\left[\omega_{i,j}+\omega_{i,j}\right]}, \end{array}$$

where *x* and *y* represent the input image, respectively. *F* is the fused images. The definition of *Q* ^*xF*^ and *Q* ^*yF*^ is the same and is given as

(17) Qi,jxF=Qg,i,jxF·Qα,i,jxF,Qi,jyF=Qg,i,jyF·Qα,i,jyF,

where *Q*_*g*_^*∗F*^ and *Q*_*α*_^*∗F*^ are the edge strength and orientation preservation values at location (*i*, *j*) of images, respectively. *∗* represents image *x* or image *y*, separately. The dynamic range for *Q* ^*AB*/*F*^ is [0,1] and it should be as close to 1 as possible for better fusion.

### 3.3. Subjective Evaluation Analysis

**Figure 3:**
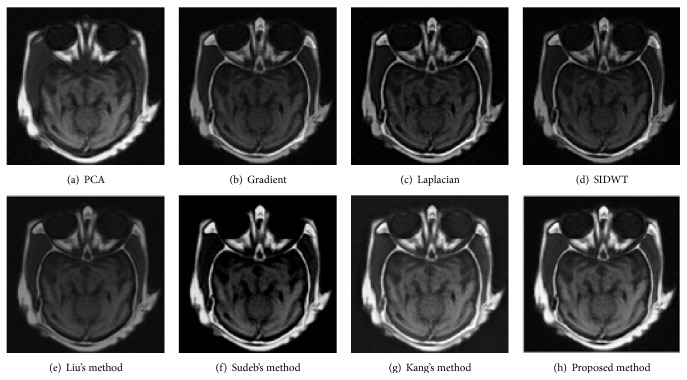
The fusion results of CT and MRI.

**Figure 4:**
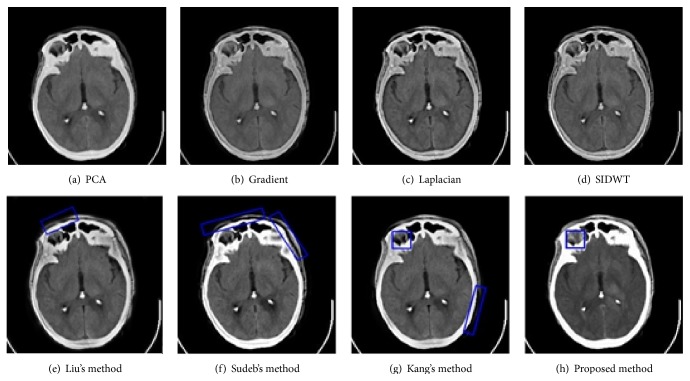
The fusion results of CT and MRI.

**Figure 5:**
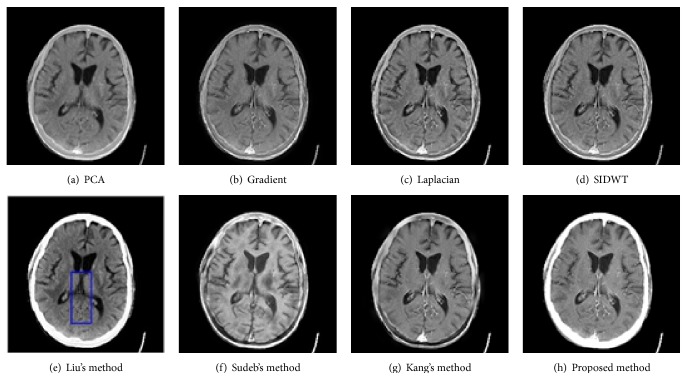
The fusion results of T1-MRI and GD-MRI.

**Figure 6:**
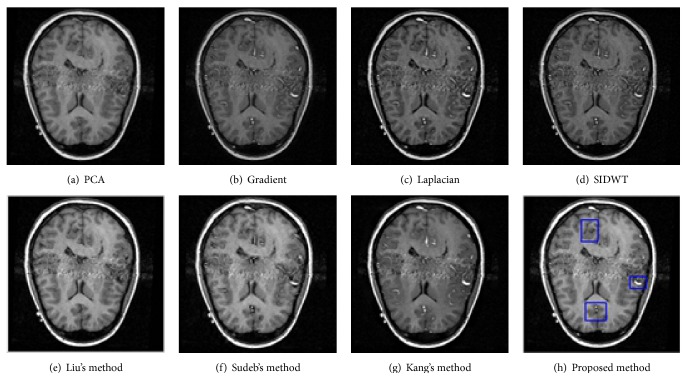
The fusion results of MRA and MRI.

**Figure 7:**
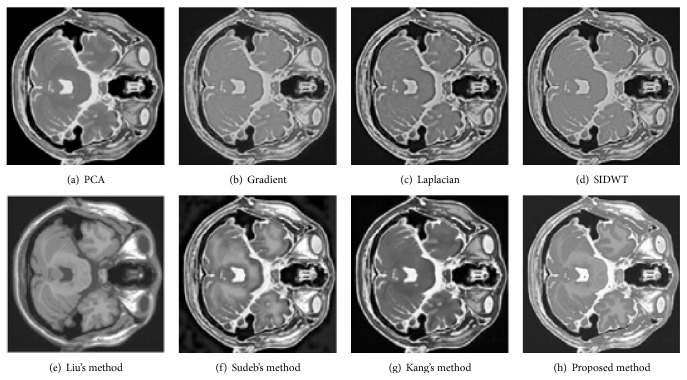
The fusion results of T1-MRI and T2-MRI.

**Figure 8:**
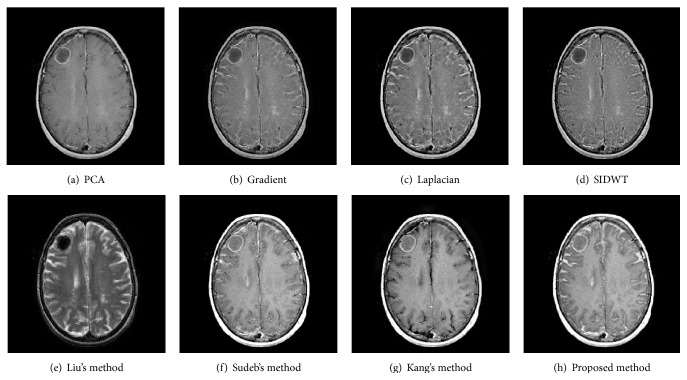
The fusion results of T1-MRI and T2-MRI.

To evaluate the performance of the proposed method in multimodal medical images fusion, extensive experiments, shown in Figures 3–8, are performed on the six groups of images, respectively. The fused images in first row of Figures 3–8 are fused results with the classic methods including PCA, Laplacian pyramid, Gradient pyramid, and SIDWT. The fused images in second row of Figures 3–8 are merged with latest three methods and proposed method. It can be clearly seen that the images fused with latest three methods and proposed method reach a higher contrast than the classic methods do in most cases. However, the proposed method is very different with the latest methods. To be specific, it can be seen that the contrast of the images fused by the presented method is higher compared to the other latest three methods by looking carefully at Figures 3(e)–3(h) and 7(e)–7(h). Figures 4(e)–4(h) demonstrate that the other three methods cannot well preserve edge information shown in the blue labeled regions of Figures 4(e)–4(g). Figures 5(e)–5(h) illustrate that Kang's method and Sudeb's method introduced many artifacts into the fused images and Liu's method lost useful information shown in the blue region in Figure 5(e). The contrast of Figure 6(g) by Kang's method is lowest among Figures 6(e)–6(h). The labeled regions by author in Figure 6(h) are clearer than the corresponding parts in Figures 6(e) and 6(f). From Figures 8(e)–8(h), it can be concluded that Liu's method is not effective to fuse images of group (f) in Figure 2 and the proposed method fuses more information from source images than Kang's method and Sudeb's method. In summary, the proposed algorithm can convert the more accurate and necessary information into the fused images than other several methods can. At the same time, less useless image information such as block effect and artifacts is introduced into the fused images by the presented scheme.

### 3.4. Objective Evaluation Analysis

Apart from the subjective performance evaluation, objective evaluation metrics are necessary to demonstrate the differences among the fused images. Tables 1, 2, and 3 demonstrate the MI and *Q* ^*AB*/*F*^ of fused images with different methods. The bold values indicate the best results in Tables 1–3. The MI value and *Q* ^*AB*/*F*^ value of the proposed algorithm are largest in the eight methods except that the *Q* ^*AB*/*F*^ values by Kang's method (Guided filter) are largest in the fusion results of group (b) and group (f). Objective evaluation results mean that the useful information converted into the fused result by the proposed algorithm is maximal among the eight approaches except special case. Moreover, the objective evaluation results of objective evaluation coincide with the visual effect evaluation very well with minor exceptions. For these exceptions, the visual effect of the proposed method is better compared to the methods with better objective evaluation performance. From above subjective performance and objective metrics comparisons, it may be concluded that the proposed algorithm can work better to combine the CT with MRI, combine the MRI with GD-MRI, combine the MRI with MRA, and combine the T1-weighted MRI with T2-weighted MRI. The proposed scheme is more effective than some state-of-the-art works and four classic methods.

## 4. Conclusion

In order to improve the effect of multimodal medical image fusion method and increase diagnostic accuracy, novel and effective medical image fusion algorithm in spatial domain is presented in this paper. The modified local contrast information is proposed as the decision map to fuse the multimodal medical images. In consideration of better global diffusion and edge-preserving ability of the adaptive manifold filter, the filtered result of source images by the adaptive manifold filter is introduced as the low-frequency part. On the other side, the modified spatial frequency of the source images is adopted as the high-frequency part. The experiment results illustrate clearly that the presented scheme is better than many other fusion methods such as guided filter method in spatial domain, NSCT-based method in transform domain, the dual-tree complex wavelet combined with the NSDFB method, and several classic image fusion methods both in subjective performance and objective evaluation.

## Figures and Tables

**Table 1:** Objective evaluation on the fusion results of groups (a) and (b).

Method	Group (a)		Group (b)	
	MI	*Q* ^*AB*/*F*^	MI	*Q* ^*AB*/*F*^
PCA	3.6627	0.6645	4.0839	0.5196
Gradient	3.2780	0.5570	3.2261	0.5218
Laplacian	2.5994	0.7085	3.1551	0.5813
SIDWT	2.9644	0.6438	3.2402	0.6097
Liu's method	5.2703	0.6454	3.8352	0.5935
Sudeb's method	4.8754	0.4563	3.7240	0.6276
Kang's method	3.4313	0.7789	3.9232	**0.8334**
Our method	** 5.8492**	**0.8022**	**4.9625**	0.6122

**Table 2:** Objective evaluation on the fusion results of groups (c) and (d).

Method	Group (c)		Group (d)	
	MI	*Q* ^*AB*/*F*^	MI	*Q* ^*AB*/*F*^
PCA	3.8985	0.4108	4.6582	0.6270
Gradient	4.0276	0.4663	3.9338	0.5628
Laplacian	4.1913	0.5368	3.5286	0.6185
SIDWT	4.1766	0.5734	3.7258	0.6047
Liu's method	3.6656	0.4579	4.2615	0.6773
Sudeb's method	3.2714	0.5374	5.0068	0.6680
Kang's method	2.9900	0.4577	3.6000	0.6230
Our method	**5.3615**	**0.5451**	**5.4437**	**0.6828**

**Table 3:** Objective evaluation on the fusion results of groups (e) and (f).

Method	Group (e)		Group (f)	
	MI	*Q* ^*AB*/*F*^	MI	*Q* ^*AB*/*F*^
PCA	5.1182	0.5772	3.6200	0.4453
Gradient	4.3344	0.5763	3.0872	0.4592
Laplacian	4.1712	0.5383	3.1039	0.5214
SIDWT	4.2474	0.4831	3.1255	0.5131
Liu's method	5.0778	0.2975	3.3798	0.3926
Sudeb's method	4.0069	0.6126	3.4720	0.5051
Kang's method	4.3699	0.2891	5.3727	**0.8883**
Our method	**8.6480**	**0.6428**	**5.5092**	0.6037
